# p38/JNK Is Required for the Proliferation and Phenotype Changes of Vascular Smooth Muscle Cells Induced by *L3MBTL4* in Essential Hypertension

**DOI:** 10.1155/2020/3123968

**Published:** 2020-12-16

**Authors:** Chaowei Hu, Kun Zuo, Kuibao Li, Yuanfeng Gao, Mulei Chen, Roumu Hu, Ye Liu, Hongjie Chi, Hongjiang Wang, Yanwen Qin, Xiaoyan Liu, Jiuchang Zhong, Jun Cai, Xinchun Yang, Jing Li

**Affiliations:** ^1^The Key Laboratory of Upper Airway Dysfunction-Related Cardiovascular Diseases, Beijing an Zhen Hospital, Capital Medical University, Beijing Institute of Heart, Lung and Blood Vessel Diseases, Beijing 100029, China; ^2^Heart Center & Beijing Key Laboratory of Hypertension, Beijing Chaoyang Hospital, Capital Medical University, Beijing 100020, China; ^3^Medical Research Center, Beijing Chaoyang Hospital, Capital Medical University, Beijing 100020, China; ^4^Hypertension Center, Fuwai Hospital, State Key Laboratory of Cardiovascular Disease of China, National Center for Cardiovascular Diseases of China, Chinese Academy of Medical Sciences and Peking Union Medical College, Beijing 100037, China

## Abstract

**Aim:**

Hypertension is a complicated disorder with multifactorial etiology and high heritability. Our previous work has identified *L3MBTL4* as a novel susceptibility gene for the development of essential hypertension, accompanied with activation of p38/JNK. Yet, little evidence has been reported whether p38/JNK contributed directly to *L3MBTL4*-induced vascular remodeling and exploring the potential mechanism of *L3MBTL4* in vascular smooth muscle cells (VSMCs).

**Methods:**

We evaluated the contribution of *L3MBTL4* on proliferation, migration, and phenotype changes of VSMCs and further explored the critical role of p38 and JNK signaling pathway underlying.

**Results:**

In *L3MBTL4* transgenic rats, we found that the elevated blood pressure, increased left ventricular hypertrophy, and thickened vascular media layer were significantly relieved by both p38 and JNK inhibitors. Meanwhile, increased cell proliferation, advanced cell cycle progression, greater migratory capability, and synthetic phenotype were observed in *L3MBTL4* overexpressed VSMCs, which could be blocked by either p38 or JNK inhibitor.

**Conclusions:**

Our findings pinpointed that p38 and JNK were required for the proliferation and phenotype changes of VSMCs induced by *L3MBTL4* in hypertension. These novel findings yield new insights into the genetic and biological basis of hypertension and are fundamental for further studies to explore the intervention strategies targeting *L3MBTL4* and p38/JNK to counteract the progression of hypertension.

## 1. Introduction

Hypertension has been the major risk factor of cardiovascular disease for years, with the overall prevalence around 30–45% [1]. It continues to be the major cause of worldwide mortality and morbidity [2, 3], with genomics proposed to have the potential to assist in reducing the overall burden of cardiovascular events [4]. Substantial progress has been made in exploring the etiology of hypertension, especially about its genetic mechanism. Several genome-wide association studies (GWAS) have identified numerous loci with >50 BP-related, single-nucleotide polymorphisms, that are associated with blood pressure (BP) regulation, which could explain the genetic tendency [5–7]. And sequencing studies have discovered novel regulatory pathways [7]. More recent successes have been made in exploring the genetic function of BP loci, such as natriuretic peptide receptor 3 (NPR3) [8] and SLC4A7 [9], which were associated with increased vascular smooth muscle cell proliferation, angiotensin II-induced calcium flux, and cell contraction [8]. Moreover, vascular smooth muscle has also been shown to be relevant to the SLC4A7 (electroneutral sodium-bicarbonate cotransporter 1) locus [9]. These results explained the contribution of BP loci to the pathogenesis of hypertension.

Of note, our previous GWAS study has identified that a genome-wide significant locus in *L3MBTL4* was strongly associated with essential hypertension and verified that *L3MBTL4* is predominantly expressed in vascular smooth muscle cells (VSMCs) and contributed to elevated BP and vascular remodeling [10]. Thus, we conferred that *L3MBTL4* gene might be a potential induce factor for hypertension. It was reported that loss, mutation, and deregulation of *L3MBTL4* was associated with human breast cancer and identified as the potential tumor suppressor gene [11]. Furthermore, *L3MBTL4* was identified as a tumor suppressor gene in myeloid malignancies and neuroblastoma either [12]. However, the potential mechanism remains uncertain especially in context of hypertension.

In recent decades, emerging evidences have demonstrated the key role of vascular remodeling throughout the pathogenesis of hypertension characterized by abnormal vascular structure, increased VSMCs proliferation and migration, and synthetic phenotype alterations [13–15]. Moreover, many studies have provided evidence for the important role of p38 and c-Jun N-terminal kinase (JNK) in the process of VSMCs proliferation, migration, and apoptosis [16, 17]. It seemed that p38/JNK was proposed participating in various vascular diseases via differential molecular mechanisms and was a new therapeutic target for treatment of vascular diseases [18]. Our previous study has found that *L3MBTL4* contributed to p38/JNK activation and vascular remodeling [10]. However, the regulatory role of *L3MBTL4* on VSMCs function and whether it was mediated by p38 and JNK remained uncertain.

Therefore, in the current work, we performed functional studies to explore the biological and cellular functions of *L3MBTL4* in VSMCs. We aimed to explore whether the blood pressure, cardiac parameters, and vascular structure in *L3MBTL4* transgenic rats (TGs) could be abolished by blocking the p38 and JNK pathways. Then, we identified the influence of *L3MBTL4* on cell proliferation, cell migration, and phenotype changes in VSMCs and explicated the potential role of the p38 and JNK signaling pathways. We confirmed that p38/JNK was required for phenotype changes of VSMCs induced by *L3MBTL4* in hypertension.

## 2. Materials and Methods

### 2.1. Animal Experiments

The research protocol was approved by the Animal Care and Use Committee of Capital Medical University, and the following animal experiments were performed based on the guideline of the Animal Ethics Committee of University. The construction and verification of *L3MBTL4* TG rats were performed in our previous study [10]. Then, we anesthetized the male rats aged 12 weeks in TGs and WTs with pentobarbital injection (120 mg/kg, i.p.), opened the thorax, got the heart, and dissected the aorta for following experiments.

Systolic BP, diastolic BP, mean BP, and heart rate of rats were measured by the noninvasive tail-cuff method and the BP-98A system (Softron, Tokyo, Japan) as previously reported [19]. Weight of different components of cardiac was measured to assess heart hypertrophy. The data were recorded by the physiological data acquisition and analyses system.

The tissues of the aorta, heart, and kidney were fixed with 4% paraformaldehyde and then embedded in paraffin and sectioned. The sections were stained with hematoxylin-eosin to determine the morphological structure of the aorta, heart, and kidney and with Masson trichrome to assess the collagen area and fibrosis severity. The measurement of media thickness, vessel diameter, media area, lumen area, and media/lumen area ratio of vasculatures at 2.5-fold and 20-fold magnification was performed using Image-Pro Plus software and the measuring tool [20]. The medial thickness of each descending aorta was obtained by calculating the mean value of 5 random measurements in different locations within the same tissue slide.

To identify the effect or JNK and p38, the specific inhibitors, SB203580 [21] and SP600125 [22], were injected to WTs and TGs. Rats were treated daily by intraperitoneal injection of 1 *µ*M SB203580/kg bodyweight in 1 ml of sterile saline or 0.01% DMSO in 1 ml of sterile saline as a vehicle control once daily for seven days. And SP600125 was prepared as a 15 mg/kg solution in a vehicle of 10% ethanol, 15% polyethoxylated castor oil (Cremophor El), 30% polyethylene glycol 400, and 20% propylene glycol (Sigma Chemical, Poole, Dorset, UK) in sterile saline. Rats were injected intraperitoneally with SP600125 (15 mg/kg) once daily for seven days.

### 2.2. Cell Experiments

Human aortic vascular smooth muscle cells were purchased from SciencCell (Carlsbad, CA, USA) and were used as VSMCs in the present work. VSMCs were cultured in smooth muscle cell medium (SciencCell) supplemented with 2% fetal bovine serum (FBS, cat #0010), 1% smooth muscle cell growth supplement (cat #1152), and 1% penicillin/streptomycin solution (P/S, cat #0503). *L3MBTL4* overexpression VSMCs were performed by transfecting pcDNA3.1 (+)_myc-His A-*L3MBTL4* (the sequence of plasmid is provided in Supplementary material 1 with lipofectamine 2000 (Invitrogen, New York, USA)). The negative controls and *L3MBTL4* overexpression plasmid were synthesized by GeneChem (Shanghai, China). In serum-free Opti-MEM1 medium, 1 *µ*g plasmid was incubated with lipofectamine 2000 for 20 minutes according to manufacturer's instructions. The mixture was further added to VSMCs. The cell culture medium was changed to DMEM containing 5% FBS. Then, we verified the *L3MBTL4* overexpression genotype by testing the expression of mRNA and protein level by qPCR (designed gene-specific primers are provided as Supplementary material 2 and Western blotting).

VSMCs were seeded in 96-well plates at a concentration of 1*∗*104 cells/well, and the cells were labeled with 10 *μ*M BrdU after starvation for 24 hours and fixed with fixing solution (BrdU proliferation assay kit, Millipore Corporation, Billerica, MA). 5 mg BrdU was dissolved with 0.5 ml 1 N NaOH, and then, distilled water was added to 5 ml. The BrdU (1000 ug/ml) storage solution would be stored in brown bottles to avoid light. Before termination of cell culture, BrdU was added (with final concentration 30 ug/L) and incubated at 37°C for 40 minutes. Subsequently, PBS washing, methanol/acetic acid fixation, 0.3% H_2_O_2_-methanol to inactivate the endogenous oxidase, 5% serum blockade, and formamide to make nucleic acid denatured (100°C for 5 min) would be performed. The anti-BrdU monoclonal antibody (rat, 1 : 50) was incubated after the washing step. Following the hematoxylin step, the total number of cells and the number of BrdU-positive cells in 10 high power fields of vision were counted randomly under the microscope to calculate the cell proliferation rate. Following the washing step, the anti-BrdU monoclonal antibody was incubated for 1 hour, and goat anti-rat IgG was conjugated. Then, peroxidase substrate was added, and the reaction was terminated by stop solution. The sample absorbance was detected by a spectrophotometer microplate reader at 450/550 nm.

Then, we measured the cell cycle progression by Cycle TEST PLUS DNA Reagent Kit. NC and *L3MBTL4* overexpression VSMCs were trypsinized, harvested, and fixed in 70% cold ethanol at 4°C. And cells were resuspended in 200 *μ*l PBS and incubated with 200 *μ*l propidium iodide at 4°C in dark after discarding the ethanol. The stained cells were filtered, and DNA fluorescence was measured by flow cytometry (BD FACS Calibur flow cytometer, Bedford, MA).

For 24-well Boyden chamber migration assay, DMEM with 10% FBS was placed in the lower layer of the transwell. Resuspended VSMCs in DMEM without FBS were added into the upper chamber at 8*∗*10^4^/well, and the cells in the 8 *μ*m-pore polycarbonate filter were fixed with 4% paraformaldehyde and stained with 0.4% crystal violet. Cells in the upper of the filter were removed, and the number of stained migrated cells was counted based on an inverted microscope (Nikon).

The specific inhibitors of JNK and p38, SB203580 (10 *μ*M), and SP600125 (10 *μ*M) were applied to *L3MBTL4* overexpression VSMCs, and the experiments mentioned above were reperformed either.

### 2.3. Western Blotting

The protein samples were extracted from VSMCs cells with the procedures essentially the same as described in details previously. As we described previously [23, 24], proteins from cultured cells were extracted with cold lysis buffer (Tris 50 mM, pH 7.4, NaCl 150 mM, Triton X-100 1%, EDTA 1 mM, and PMSF 2 mM). The supernatant was harvested after centrifugation, and the concentration of protein was determined by using the BioRad protein assay kit (BioRad Laboratories, Inc., Berkeley, CA, USA). Then, 20 *µ*g protein samples were electrophoresed on 8%–10% SDS-PAGE and transferred onto nitrocellulose membrane (Millipore, USA), which would be blocked with 5% nonfat milk in Tris-buffered saline buffer (20 mM Tris, 150 mM NaCl, pH 7.6, Tween20: 0.1%). And the membranes were incubated with primary antibodies at 4°C overnight and reacted with appropriate secondary antibodies. Subsequently, the blot bands would be developed by enhanced chemiluminescence reagents (Amersham, UK) and detected by the BioRad imaging system where the band signal intensities were quantitatively analyzed by Quantity One software. The antibodies against *L3MBTL4* (Novus, Catalog #NBP2-15009, rabbit, polyclonal antibody), phosphor JNK (Abcam, Catalog #ab131499, rabbit, polyclonal antibody), nonphosphor JNK (Beyotime, Catalog #AJ518, rabbit, polyclonal antibody), phosphor p38 (Beyotime, Catalog #AM063, mouse, monoclonal antibody), nonphosphor p38 (Beyotime, Catalog #AF1111, rabbit, monoclonal antibody), PCNA (Beyotime, Catalog #AF0261, mouse, monoclonal antibody), cyclin *A* (Boster, Catalog #PB0402, rabbit, polyclonal antibody), cyclin *D* (Beyotime, Catalog #AC853-1, mouse, monoclonal antibody), p21 (Beyotime, Catalog #AP021-1, mouse, monoclonal antibody), p27 (Beyotime, Catalog #AP027-1, mouse, monoclonal antibody), MMP-2 (Abcam, Catalog #ab97779, rabbit, polyclonal antibody), MMP-9 (Abcam, Catalog #ab38898, rabbit, polyclonal antibody), TIMP-1 (Abcam, Catalog #ab211926, rabbit, monoclonal antibody), TIMP-2 (Abcam, Catalog #ab53730, rabbit, polyclonal antibody), SM-22*α* (OriGene, Catalog #TA503092, mouse, monoclonal antibody), and OPN (Boster, Catalog #PB0589, rabbit, polyclonal antibody) were used, with *β*-actin (Affinity, Catalog #BF0198, mouse, monoclonal antibody) as internal controls. All the target protein band intensity data in our present study have been normalized in relation to the internal loading control, and the phosphosignals have been normalized to the total band intensities.

### 2.4. Immunohistochemical Staining

For immunohistochemical staining with positive cells of Ki67, alpha smooth muscle actin, smooth muscle myosin heavy chain, and desmin, paraffin-embedded sections of tissue underwent antigen retrieval by incubation in citrate buffer for 5 min at 85°C. Following 5% normal goat serum incubation, the sections were treated with antibodies specific to Ki67 (GB111141, Servicebio), alpha smooth muscle actin (GB111364, Servicebio), smooth muscle myosin heavy chain (GB11805, Servicebio), and desmin (GB11081, Servicebio) overnight at 4°C. After incubated with secondary antibodies for 1 h at room temperature, the section samples were stained with avidin-biotin complex and counterstained with hematoxylin. For TUNEL assay, the TdT-mediated dUTP nick end labeling kit (GDP1041, Servicebio) was performed in the aortic tissue to determine TUNEL-positive staining. The stainings were examined, and images were captured under a microscope (Nikon).

### 2.5. Statistical Analysis

Quantitative data were presented as mean ± s.e.m. Student's *t*-test and Dunnett's test were used for group comparisons. *P* value < 0.05 was regarded as significant.

## 3. Results

### 3.1. p38 and JNK Were Essential for the Impact of *L3MBTL4* on Blood Pressure, Left Ventricular Hypertrophy, and Vascular Remodeling

Our previous study has identified that the p38/JNK pathway was activated in *L3MBTL4* TGs [10], but it still remained unclear whether the p38/JNK pathway contributed directly to *L3MBTL4*-induced vascular remodeling and whether inhibition of the p38/JNK pathway could abolish the role of *L3MBTL4*. Therefore, we applied p38 specific inhibitor, SB203580 [21], and JNK specific inhibitor, SP600125 [22], in the current study to explore the role of p38/JNK during the process of *L3MBTL4*-induced elevated blood pressure, left ventricular (LV) hypertrophy, and vascular remodeling in hypertension.

**Figure 1:**
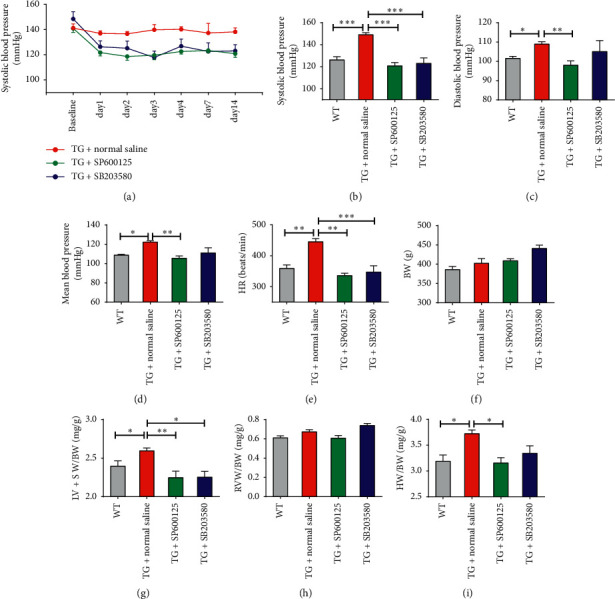
p38/JNK was required for elevated blood pressure and left ventricular hypertrophy mediated by *L3MBTL4*. (a) The systolic blood pressure (SBP) in TGs, compared with TG + SP600125 and TG + SB2003580. (b)–(f) SBP, diastolic blood pressure (DBP), mean blood pressure (MBP), heart rate (HR), and bodyweight (BW) of WTs, TG + normal saline, TG + SP600125, and TG + SB2003580 (*n* = 5 each group). (g)–(i) Quantitative analysis of left ventricle + septum weight/bodyweight (LV + SW/BW), right ventricle weight/bodyweight (RVW/BW), and heart weight/bodyweight (HW/BW) ratios in each group (*n* = 5 each group). ^*∗*^*p* < 0.05; ^*∗∗*^*p* < 0.01; ^*∗∗∗*^*p* < 0.001. All data represent mean ± SEM.

At first, we detected whether p38/JNK was necessary during the process of *L3MBTL4*-mediated elevated blood pressure and LV hypertrophy. We found that the effects of *L3MBTL4*-mediated higher systolic BP (Figures 1(a) and 1(b), diastolic BP (Figure 1(c)), mean BP (Figure 1(d)), and heart rhythm (Figure 1(e)) in TGs were abolished by p38/JNK inhibitor. When SB203580 or SP600125 was applied, the elevated BP showed the declined trend. Besides the directly elevated BP, cardiac hypertrophy is also the phenotype of hypertension and has been suggested as the hypertension-related target organ damage. As the results showed, the elevated left ventricle and septum weight/BW ratio (LV + SV/BW) (Figure 1(g)) and heart weight/BW ratio (HW/BW) (Figure 1(i)) in TGs were relieved by p38/JNK inhibitor similarly. So far, it followed that pathological analysis of heart morphology revealed myocardium remodeling induced by *L3MBTL4*, with pathological hypertrophy and myocardial fibrosis. And p38 and JNK inhibitors lead to attenuated myocardial hypertrophy and perivascular and interstitial fibrosis in the heart (Figures S1(a) and S1(b).

**Figure 2:**
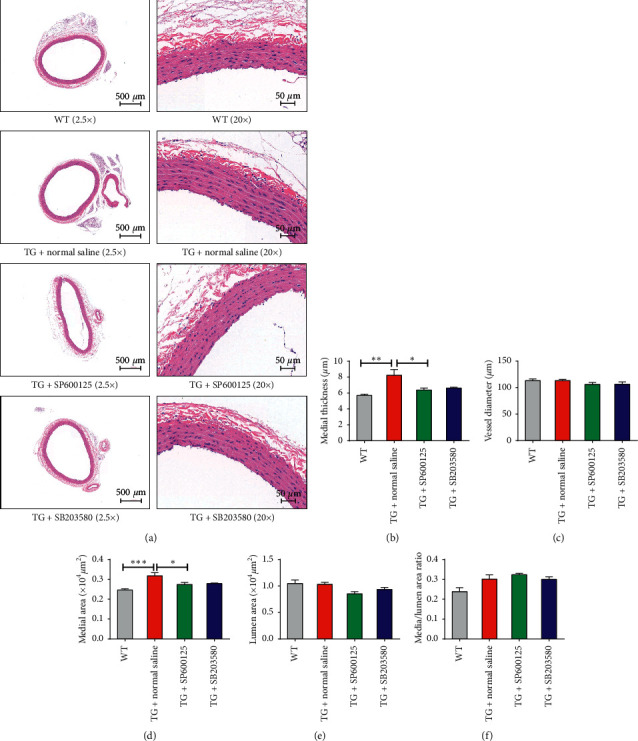
p38/JNK was required for vascular remodeling mediated by *L3MBTL4*. (a) Representative photomicrographs of hematoxylin-eosin staining in blood vessels from WTs, TG + normal saline, TG + SP600125, and TG + SB2003580 (*n* = 5 each group). (b)–(f) The media thickness, vessel diameter, media area, lumen area, and media/lumen area ratio of aortas in each group was quantified (*n* = 5 per group). The magnification was 2.5-fold and 20-fold. Scale bars are 50 *μ*m and 10 *μ*m. ^*∗*^*p* < 0.05; ^*∗∗*^*p* < 0.01; ^*∗∗∗*^*p* < 0.001. All data represent mean ± SEM.

Then, we observed the role of p38/JNK inhibitor in *L3MBTL4*-mediated vascular remodeling. Vascular remodeling was the key structural alteration and significant pathophysiological change in hypertension. The effect of the thickened media layer of blood vessel (Figure 2(a) and 2(b), a higher media/lumen area ratio (Figure 2(d)) mediated by *L3MBTL4* in TGs was mitigated by p38/JNK inhibitors. Furthermore, to examine the histology of the kidney, we performed hematoxylin-eosin staining of renal tissue. Small arteries with thicker vascular wall and glomeruli without significant changes in the kidney were observed in *L3MBTL4* TGs (Figure S1(c)). The glomeruli was unaffected under p38 and JNK inhibitor treatment (Figure S1(c)). And it was found that the pathological alternations of small arteries were slightly improved by p38 and JNK inhibitors (Figure S1(d)).

### 3.2. p38/JNK Was Required for VSMCs Proliferation Mediated by *L3MBTL4*

According to the pivotal role of p38/JNK in *L3MBTL4*-mediated elevated BP, left ventricular hypertrophy, and vascular remodeling, we further focused on VSMCs, the most prominent cellular constituent of blood vessel. The proliferation of VSMCs contributed to increased intima-media thickness, arterial stiffness, and blood pressure and has been suggested as the critical determinant of vascular disease [14].

**Figure 3:**
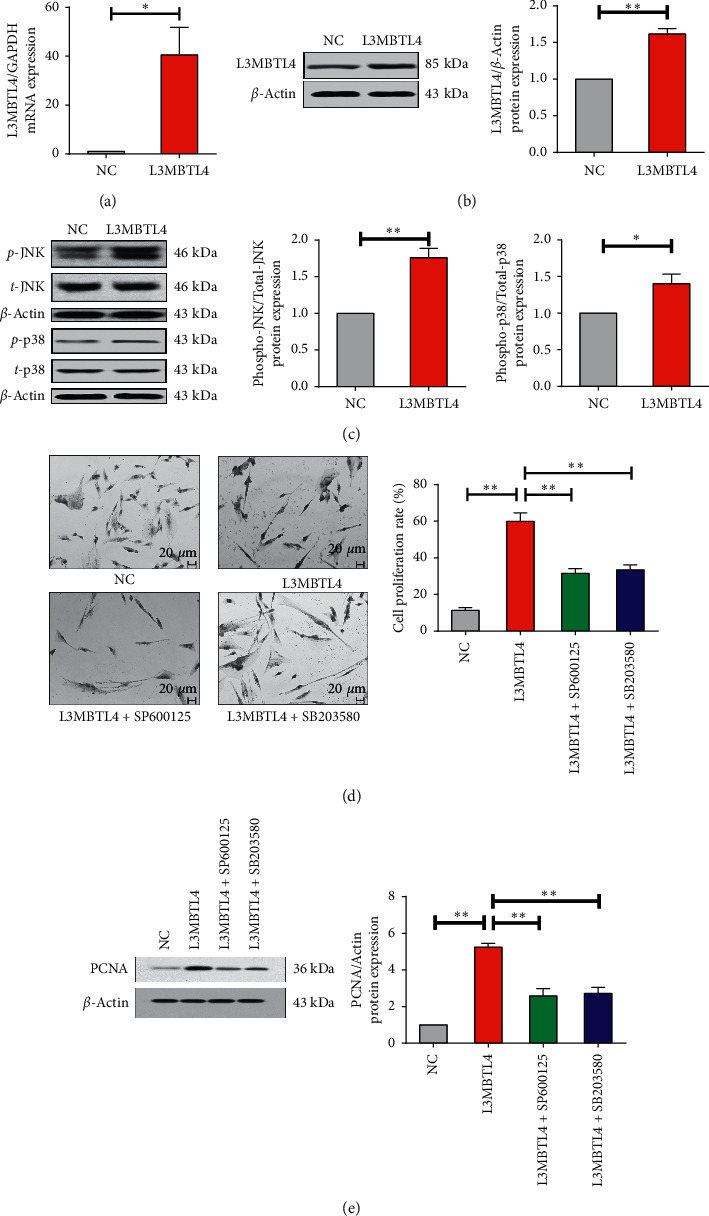
p38/JNK was required for VSMCs proliferation mediated by *L3MBTL4*. (a) Relative mRNA expression of *L3MBTL4* in negative control (NC) and *L3MBTL4* overexpression VSMCs. (b) L3MBTL4 protein levels in NC and *L3MBTL4* overexpression VSMCs were measured by Western blot. (c) Western blot analysis validated the phosphorylation levels of p38 and c-Jun *N*-terminal kinase (JNK) in NC and *L3MBTL4* overexpression VSMCs. (d) Proliferation of VSMCs in NC, *L3MBTL4* overexpression VSMCs, *L3MBTL4* overexpression VSMCs + SP600125, and *L3MBTL4* overexpression VSMCs + SB2003580 was measured by BrdU incorporation assay. (e) PCNA expression in each group was determined by Western blot (*n* = 3). ^*∗*^*p* < 0.05; ^*∗∗*^*p* < 0.01. All data represent mean ± SEM.

To determine the proliferative activity and apoptotic status of smooth muscle cells in aortic tissue, Ki67 and TUNEL staining were performed, respectively. It was detected that *L3MBTL4* elicited neither alternation of Ki67 proliferating cells or TUNEL-positive cells in vasculature, which was not affected by p38/JNK inhibitors administration (Figure S2). Nevertheless, to investigate the potential role of *L3MBTL4* during the process of cell proliferation and migration, we transfected specific *L3MBTL4* expression vector into VSMCs and verified the transfection effect on both mRNA and the protein level by qPCR and Western blotting (Figure 3(a) and 3(b). Then, we validated the elevating protein expression of phosphor-p38 and JNK (Figure 3(c)) and reconfirmed the phosphorylated activating effect mediated by *L3MBTL4* in *L3MBTL4* overexpression VSMCs.

**Figure 4:**
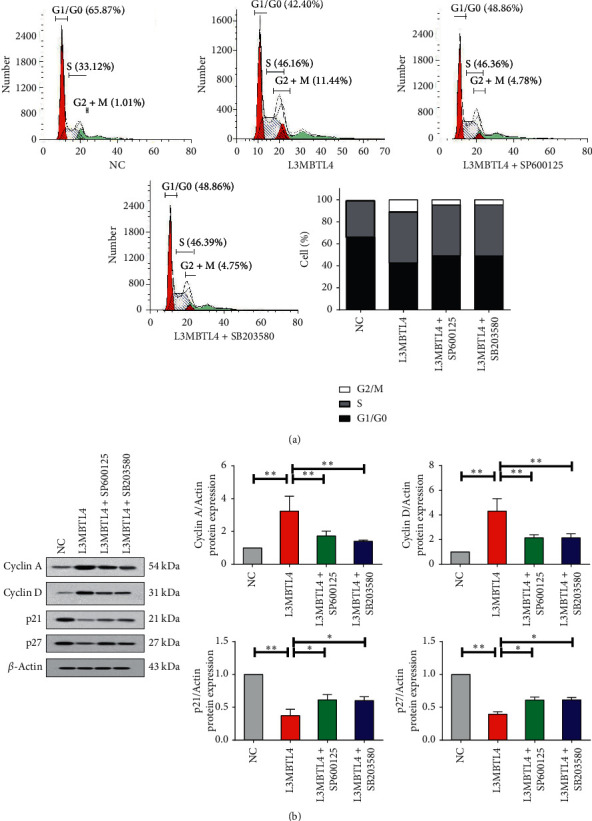
p38/JNK was required for propelled cell cycle mediated by *L3MBTL4*. (a) The percentage of cells in G0/*G*1, *S*, and G2/M phases of cell cycle in NC, *L3MBTL4* overexpression VSMCs, *L3MBTL4* overexpression VSMCs + SP600125, and *L3MBTL4* overexpression VSMCs + SB2003580 was quantified by flow cytometer analysis (*n* = 3). (b) Western blot analysis of cyclin *A*, cyclin *D*, p21, and p27 expression (*n* = 3). ^*∗*^*p* < 0.05; ^*∗∗*^*p* < 0.01; ^*∗∗∗*^*p* < 0.001.

We measured cell proliferation by BrdU incorporation assay and found that *L3MBTL4* overexpression led to increased cell proliferation (Figure 3(d)). Consistently, VSMCs with *L3MBTL4* overexpression exhibited enhanced expression of proliferation cell nuclear antigen (PCNA) [25] (Figure 3(e)). Furthermore, we evaluated the effect of *L3MBTL4* on cell cycle, and the results from flow cytometer analysis indicated that with the overexpression of *L3MBTL4*, the percentage of *S* phase cell was remarkably increased from 33.12% to 46.16%, accompanied by decreased proportion of *G*0/*G*1 phase from 65.87% to 42.40% (Figure 4(a)). Meanwhile, we noted that upon *L3MBTL4* overexpression, levels of cell cycle-related proteins cyclin *A* and cyclin *D* were strikingly elevated, which was in accordance with decreased cyclin-dependent kinase inhibitors p21 and p27 (Figure 4(b)).

We next evaluated the impact of p38 and JNK kinase inhibition on VSMCs proliferation. Either SB203580 or SP600125 succeeds to inhibit the promoting effect mediated by *L3MBTL4*, displayed by decreased cell proliferation rate and expression of PCNA (Figures 3(d) and 3(e)). Consistently, p38/JNK inhibitors significantly blocked the accelerated cell cycle, manifested with decreased cell percentage of *S* phase, expression of cyclin *A* and cyclin *D,* and repicked up p21 and p27 [26] (Figures 4(a) and 4(b)). It appeared that p38/JNK was necessary for *L3MBTL4* to play the role of the proliferation-promoting effect caused by the accelerated cell cycle.

Furthermore, the matrix metalloproteinases (MMPs) and tissue inhibitors of metalloproteinases (TIMPs) have widely accepted implications in matrix remodeling in the aorta and pulmonary artery in relation to hypertension [27]. TIMP-1 and TIMP-2 binds to pro-MMP-9 and pro-MMP-2, respectively, and serve as inhibitors of MMP-2 and MMP-9 [28]. Therefore, the protein expressions of MMP-2, MMP-9, TIMP-1, and TIMP-2 were determined to evaluate the protective effect of p38 and JNK inhibitors in status of vascular remodeling induced by *L3MBTL4*. The results showed that both the protein levels of MMP-2 and MMP-9 are significantly enhanced by *L3MBTL4*, which are partly abolished by blocking p38 and JNK. While TIMP-1 and TIMP-2, which serve as inhibitors of MMP-2 and MMP-9, showed completely inverse variation to MMP-2 and MMP-9. These results indicate that *L3MBTL4* and its downstream signaling pathways, p38 and JNK, promote vascular remodeling and proliferation of vascular smooth muscle cells partly through TIMP-1/MMP-9 and TIMP-2/MMP-2 (Figure S3).

### 3.3. p38/JNK Was Required for Migration and Phenotype Alteration of VSMCs Mediated by *L3MBTL4*

**Figure 5:**
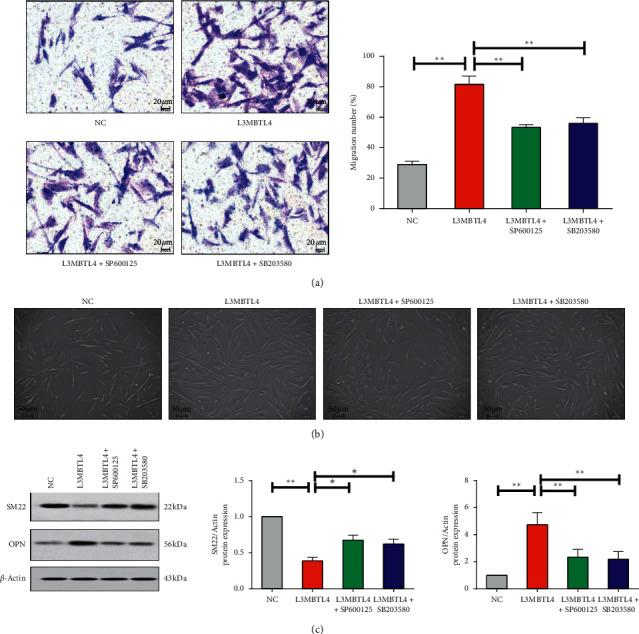
p38/JNK was required for VSMCs migration mediated by *L3MBTL4*. (a) Boyden chamber migration assays were performed, and the number of migratory VSMCs was assessed by crystal violet staining (*n* = 3). (b) Representative photomicrographs of cell morphology in the confluent cultured dishes of negative control, *L3MBTL4* overexpressed cells, and cells treated with inhibitors. Scale bars are as shown. (c) Protein levels of SM-22 and OPN were measured by Western blot (*n* = 3). ^*∗*^*p* < 0.05; ^*∗∗*^*p* < 0.01; ^*∗∗∗*^*p* < 0.001.

As we know, with the excessive cell proliferation, VSMCs will migrate to intima and cause thickened vascular wall and enhanced vascular resistance, which contribute to the progression of hypertension simultaneously [14]. Accordingly, we examined cell migration by Boyden chamber migration assay and detected that *L3MBTL4* overexpression induced higher number of migrated VSMCs, which suggested a greater migratory capability induced by *L3MBTL4* (Figure 5(a)). So far, these results confirmed the pivotal role of p38/JNK in *L3MBTL4-*mediated VSMC migration.

We next evaluated the effect of *L3MBTL4* in VSMCs phenotype changes, another mechanism involving the progression of hypertension, characterized by VSMCs switching from contractile to synthetic phenotype, and accompanied by increased synthetic protein osteopontin (OPN) and decreased smooth muscle contractile protein SM-22*α* [29–31]. The cultured VSMCs in the confluent cultured dishes are shown in Figure 5(b) to examine cell phenotype modulations. The control VSMCs were spindle-shaped in morphology, and most *L3MBTL4* overexpressed cells were fibroblastic in appearance. The majority of VSMCs treated with inhibitors reverted to the control morphology. Moreover, our results showed that the expression of OPN was remarkably upregulated, followed by downregulated SM-22*α* at the protein level. The p38 inhibitor SB203580 and JNK inhibitor SP600125 suppressed VSMCs migration similarly (Figure 5(c)). These data, taken together, demonstrated that p38/JNK was essential for phenotype changes induced by *L3MBTL4*.

In addition, to identify the expression of VSMC differentiation markers, we performed immunohistochemical staining of alpha smooth muscle actin, smooth muscle myosin heavy chain, and desmin in blood vessel. It was observed that these VSMCs differentiation markers were reduced in the aorta from *L3MBTL4* TGs, but more abundant in rats treated with p38 and JNK inhibitors, indicating a transformation of VSMCs from synthetic to contractile phenotype by blocking p38 and JNK (Figure S4).

## 4. Discussion

For the purpose of identifying novel variants contributed to hypertension, our previous genome-wide association study was performed and pinpointed *L3MBTL4* as the new gene significantly associated with hypertension. Then, we identified that *L3MBTL4* is predominantly expressed in VSMCs, and *L3MBTL4* TGs exhibited remarkable elevated BP, vascular remodeling, and cardiac hypertrophy [10]. Based on these findings, we further identified that *L3MBTL4* leads the higher BP, LV hypertrophy, and thickened vascular media layer, by conducing to increased cell proliferation, advanced cell cycle progression, greater migratory capability, and synthetic phenotype alterations in VSMCs. What is more, we identified that phosphorylation of p38/JNK is the pivotal step mediating *L3MBTL4*-induced hypertension. According to the results from application of inhibitors of p38/JNK, we found that p38/JNK was required for *L3MBTL4* to play the role in promoting the progression of hypertension and propelling VSMCs proliferation and phenotype alteration. In general, we explicated the specific mechanism of *L3MBTL4* to regulate blood pressure, which is induced by the p38/JNK pathway and characterized by promoting VSMCs proliferation, migration, and switching from contractile to synthetic phenotype.

Moreover, hypertension is characterized by disordered vascular function and structure. The arterial vascular wall can be divided into 3 layers: intima, media, and externa, which contain multiple cellular components such as endothelial cells, VSMCs, and extracellular matrix. VSMCs are the dominant cellular constituents of arteries and the critical determinant of vascular disease. VSMCs proliferation is closely linked to vascular remodeling and hypertension, which results in an increased intima-media thickness and contributes to increased arterial stiffness and blood pressure [14]. In addition, VSMCs migration is a normal process that occurs during vascular development or for tissue repair in response to vascular injury. However, pathological migration is a major factor in maladaptive vascular remodeling and pivotal to extensive intimal thickening [15]. Furthermore, VSMCs may preserve phenotype alterations from contractile (differentiated) type to synthetic (dedifferentiated) type, while this transformation is involved in dysregulation expression of reduced contractile proteins and increased extracellular matrix and may serve as the major factor for vascular remodeling [32, 33]. Consistent with our results, overexpression of hypertension susceptibility gene *L3MBTL4* promoted the VSMCs proliferation, migration, and transformation to synthetic type.

Meanwhile, we analyzed the potential signaling pathway involved in the process. We noticed remarkable phosphorylation activation of p38/JNK in the *L3MBTL4* overexpression model. It was previously found that mean arterial pressure of sham control mice was not affected by JNK inhibitor SP600125 [34]. In addition, it was demonstrated that p38 MAPK inhibitor SB203580 did not affect normal blood pressure [35]. And researchers recently identified SB203580 to have no effect in the impaired acetylcholine-induced relaxation of WKY rats (normal rats) [36]. Therefore, it is suggested that p38/JNK inhibitors have hardly an effect on the blood pressure of normal rats, as p38/JNK may not be activated in the baseline state. The inhibitor of p38/JNK could abolish the proliferation, migration, and phenotype changes-promoting effect induced by *L3MBTL4*. A series studies had revealed that activation of the p38/JNK pathway appeared to be the key event associated with VSMCs function and determining whether a human microvascular endothelial cell survives or undergoes programmed cell death [37]. Alkaloid rich fraction targets VSMC proliferation and migration to attenuate neointima formation by inhibition of PDGF-R*β*, p38, and JNK-mediated signaling [38]. In addition, it has been revealed that cyclic mechanical stretch of rat aortic VSMCs caused JNK- and p38-dependent cell death and JNK and p38 inhibitors decreased cell death, which may have a clinical value in aortic dissection caused by acute rise in blood pressure [39]. Moreover, previously investigators have suggested a potential correlation of TIMP-1/MMP-9 and TIMP-2/MMP-2, with p38 and JNK in various cell types. p38 signaling is identified to be necessary for MMP-2 secretion and activation in MC3T3-E1 cells [40], and the specific p38 inhibitor SB203580 was found to inhibit the expression of MMP-2 in rat lung fibroblasts [41]. Both MMP-2 and MMP-9 in nonsmall cell lung cancer cells were indicated to be downstream effectors of p38 signaling pathways [42]. The downregulation of p38 MAPK and JNK was shown to result in decreased MMP-2 and MMP-9 expressions in human lung adenocarcinoma cells [43]. Most recent study has demonstrated JNK inhibitor to reduce EGF-mediated MMP-9/TIMP-1 ratio in HTR-8/SVneo trophoblastic cells [44]. The significant implication of our present results is that p38/JNK is required for proliferation, migration, and phenotype changes of VSMCs induced by *L3MBTL4* in hypertension.

## 5. Conclusion

The present study characterized that p38/JNK was required for the proliferation and phenotype alteration of VSMCs mediated by *L3MBTL4* in hypertension. These novel findings yield new insights into the genetic and biological basis of hypertension and are fundamental for further studies to explore the intervention strategies targeting *L3MBTL4* and p38/JNK to counteract the progression of hypertension.

## Data Availability

The data used to support the findings of this study may be released upon application to the Beijing Chaoyang Hospital that can be contacted at yxc6229@163.com.

## Supplementary Materials

Supplementary Materials Supplementary material 1: sequence of plasmid pcDNA3.1(+)_myc-His A- *L3MBTL4*. Supplementary material 2: designed gene-specific primers. Figure S1: pathological changes of the myocardium and kidney in *L3MBTL4* TGs were improved when inhibiting p38 and JNK. Figure S2: p38/JNK was essential for elevated proliferative activity and depressed apoptotic status of smooth muscle cells in aortic tissue. Figure S3: *L3MBTL4* and its downstream p38 and JNK were partly mediated by TIMP-1/ MMP-9 and TIMP-2/MMP-2. Figure S4: p38/JNK inhibition lead to improved expression of VSMCs differentiation markers in *L3MBTL4* TGs.
