# Supplementary material for: p38/JNK Is Required for the Proliferation and Phenotype Changes of Vascular Smooth Muscle Cells Induced by L3MBTL4 in Essential Hypertension
Source: Int J Hypertens. 2020 Dec 16;2020:3123968. doi: 10.1155/2020/3123968 (PMC7759026; doi:10.1155/2020/3123968)
Supplement: Supplementary Materials — Supplementary material 1: sequence of plasmid pcDNA3.1(+)_myc-His A- L3MBTL4. Supplementary material 2: designed gene-specific primers. Figure S1: pathological changes of the myocardium and kidney in L3MBTL4 TGs were improved when inhibiting p38 and JNK. Figure S2: p38/JNK was essential for elevated proliferative activity and depressed apoptotic status of smooth muscle cells in aortic tissue. Figure S3: L3MBTL4 and its downstream p38 and JNK were partly mediated by TIMP-1/ MMP-9 and TIMP-2/MMP-2. Figure S4: p38/JNK inhibition lead to improved expression of VSMCs differentiation markers in L3MBTL4 TGs. [file 3123968.f1.zip › 3123968.f1/Supplemenatry Materials 1 and 2.docx]

**Additional files**

**Additional files 1: Sequence of plasmid pcDNA3.1(+)_myc-His A-L3MBTL4.**

GAATTCATGAAACAGCCCAACAGGAAAAGGAAGCTTAATATGGATTCCAAAGAGCGTTTGGATCAGGACGGACGCTTGGAGCAAGCTGAAGAGGAAAAGAAGCCCAAGGATAGCACAACCCCTTTGAGTCACGTCCCTTCAGCGGCTGCACAGGGAGCATGGTCTTGGGAGTGGTACTTGAAAGAACAGAAGGCTGTCGCAGCACCTGTTGAGCTGTTTTCCAAGGATCAGTCCTTTCCAGAGCATGAAAATGGTTTTCAGATTGGAATGAGATTAGAAGGCATTGATCCCCGACATCCATCGGTATTCTGTGTGCTTTCTGTAGCGGAGGTTTGTGGTTACCGTCTAAGACTTCATTTTGATGGTTATTTAAGTTGCTATGATTTTTGGACCAATGCTGGTTCCCCTGACATTCATCCAGTAGGATGGTGTGAAAAGACCAAACATGAACTGCACATCCCTAAGGGTTATAGAAAAGATAAATTTGTTTGGATGGATTACTTGAAGGCCTGCAAATTGCAAAATGCTCCAAAGAAATTATTCAGAAACAGAAGTCCTAATGGGCCAATGTCTAAAGAATTTCAGGTTGGAATGAAGCTGGAGGCCGTGGACAGGAAGAACCCTTCCTTGGTGTGTGTGGCGACCATAGCAGATATTGTTGAAGATCGCTTACTAGTGCATTTTGACAACTGGGATGATAGTTACGATTACTGGTGCGATGTTAATAGCCCTTATGTCCAGCCAGTTGGTTGGTGTCAGGAGAATGGAAGAACTCTGATAGCACCCCAAGGTTATCCCAATCCAGAAAATTTTTCCTGGACAGAATACCTGGAAGCTACTCAAACCAATGCAGTTCCTGCCAAAGTTTTTAAAATGAGGTTGCCTCATGGTTTTCTGCCAAATATGAAACTTGAAGTTGTGGATAAACGGAACCCCAGGTTAATTCGTGTTGCTACGATTGTAGATGTTGATGACCAAAGAGTAAAGGTTCATTTTGATGGTTGGGACCATAAGTATGACTACTGGGTGGAGGCAGACAGCCCTGATATCCACCCGATCGGATGGTGTGATGTCACAGGGCATCCACTGGAAGTGCCACAGCGAACGAATGACCTGAAGATCCTTCCAGGTCAAGCTGTCTGTCCTACTCCCGGGTGCCGAGGAATAGGCCATATCCGTGGTCCACGTTATTCGGGACATCACAGTGCTTTTGGCTGCCCGTATTCAGACATGAACTTGAAAAAGGAGGCAACACTTCACGATCGTTTGAGAGAACAAACACAGGCAAATTTGGAATCAGACTCTTCACATTCAAAATCAAAAAGCCTCTGTAGTTTGAACTTCAATGGAAAACATGAAAAGGTGAACAGTCAGCCCAGACTTGTACAGCAGGCAAAGTGTTTGAAAATCAAAGGAAAAGAAGATATTGACTTGGATAATCTCTTCAGAGAATACTCGGTGGAGCAGGCGCAGCAGGTGCTTCACCAGTCAGTGTCCATGTCCACGGTGTCAGCCCACCCTTTTCGGGACCTTCCCCTCGGCAGGGAGCAACACTGCAAGTTGCTTCCAGGCGTGGCTGACATCCGGGCCAGCCAAGTGGCACGTTGGACTGTGGATGAGGTGGCTGAGTTTGTACAGTCTCTTCTGGGCTGTGAAGAGCATGCCAAGTGCTTTAAGAAAGAGCAGATCGATGGCAAAGCCTTCCTGCTTCTGACACAGACGGACATTGTCAAAGTGATGAAGATCAAACTGGGCCCAGCACTGAAGATTTACAACTCTATCCTGATGTTCAGGCATTCCCAGGAACTCCCTGAAGAAGATATTGCCTCAGGCCAAGAAGTCAGGGGAGGATCC

**Additional files 2: Designed gene-specific primers:**

L3MBTL4-F: TAGCCCTTATGTCCAGCCAG,

L3MBTL4-R: GGGTTCCGTTTATCCACAAC,

GAPDH-F: GCCTTCCGTGTCCCCACTGC,

GAPDH-R: GGCTGGTGGTCCAGGGGTCT
