# Supplementary figures and images for: p38/JNK Is Required for the Proliferation and Phenotype Changes of Vascular Smooth Muscle Cells Induced by L3MBTL4 in Essential Hypertension
Source: Int J Hypertens. 2020 Dec 16;2020:3123968. doi: 10.1155/2020/3123968 (PMC7759026; doi:10.1155/2020/3123968)

**a**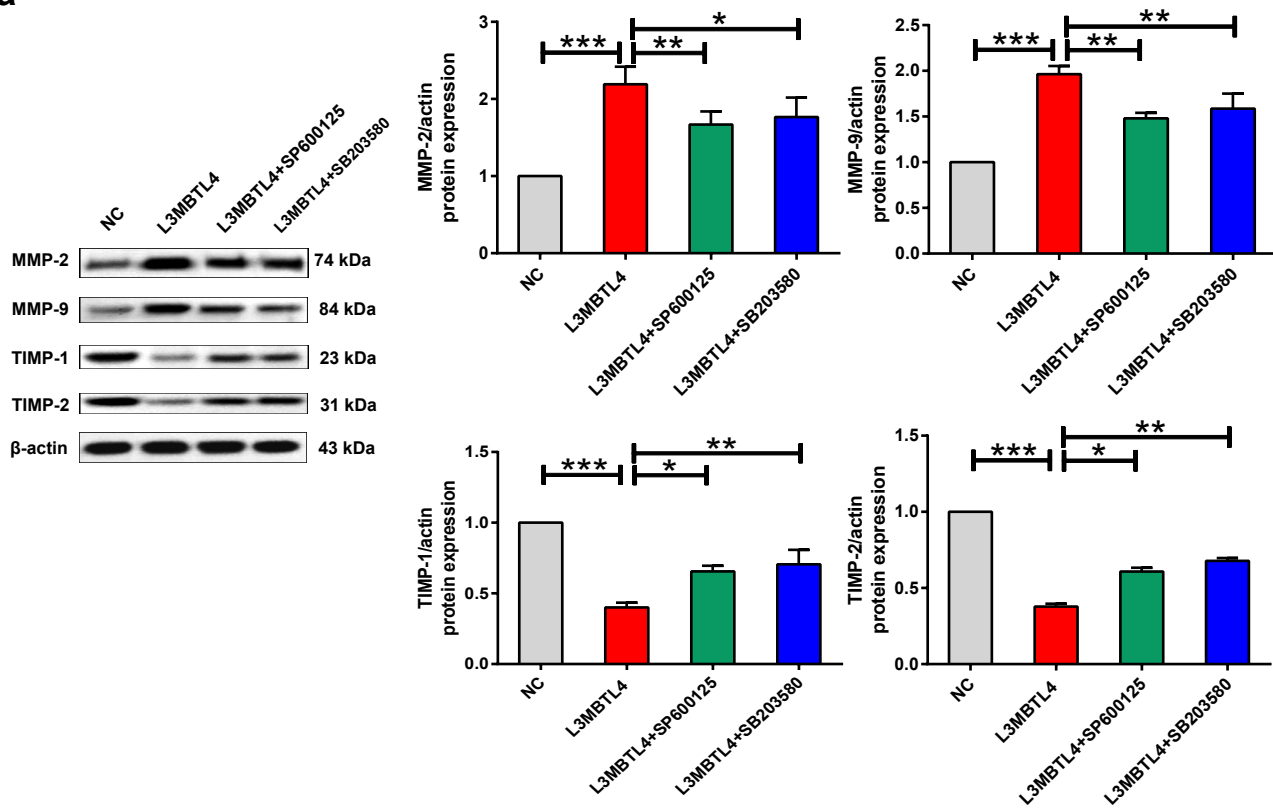

Supplement: Supplementary Materials — Supplementary material 1: sequence of plasmid pcDNA3.1(+)_myc-His A- L3MBTL4. Supplementary material 2: designed gene-specific primers. Figure S1: pathological changes of the myocardium and kidney in L3MBTL4 TGs were improved when inhibiting p38 and JNK. Figure S2: p38/JNK was essential for elevated proliferative activity and depressed apoptotic status of smooth muscle cells in aortic tissue. Figure S3: L3MBTL4 and its downstream p38 and JNK were partly mediated by TIMP-1/ MMP-9 and TIMP-2/MMP-2. Figure S4: p38/JNK inhibition lead to improved expression of VSMCs differentiation markers in L3MBTL4 TGs. [file 3123968.f1.zip › 3123968.f1/FigureS3.pdf]

**a** TUNEL

WT

TG+Normal Saline

100μm  
I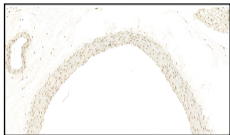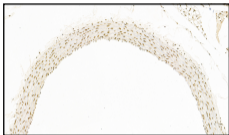

TG+Sp600125

TG+SB203580

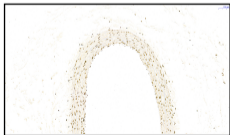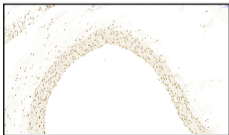**b** Ki67

WT

TG+Normal Saline

100μm  
I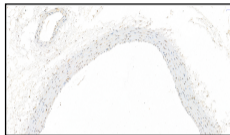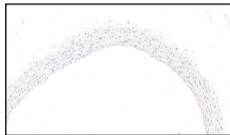

TG+Sp600125

TG+SB203580

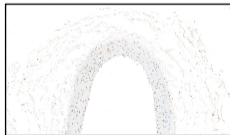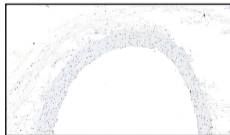

Supplement: Supplementary Materials — Supplementary material 1: sequence of plasmid pcDNA3.1(+)_myc-His A- L3MBTL4. Supplementary material 2: designed gene-specific primers. Figure S1: pathological changes of the myocardium and kidney in L3MBTL4 TGs were improved when inhibiting p38 and JNK. Figure S2: p38/JNK was essential for elevated proliferative activity and depressed apoptotic status of smooth muscle cells in aortic tissue. Figure S3: L3MBTL4 and its downstream p38 and JNK were partly mediated by TIMP-1/ MMP-9 and TIMP-2/MMP-2. Figure S4: p38/JNK inhibition lead to improved expression of VSMCs differentiation markers in L3MBTL4 TGs. [file 3123968.f1.zip › 3123968.f1/R1-FigureS2.pdf]

**a** alpha smooth muscle Actin

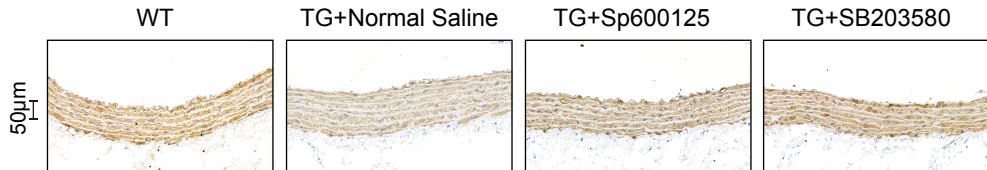

**b** smooth muscle Myosin heavy chain

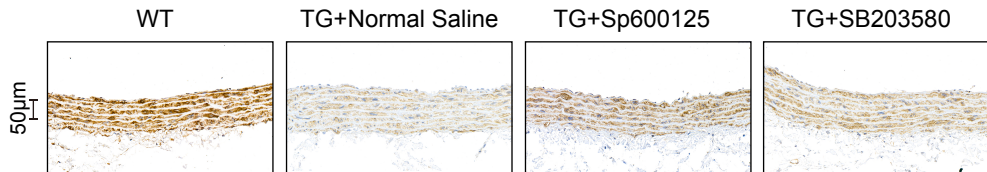

**c** Desmin

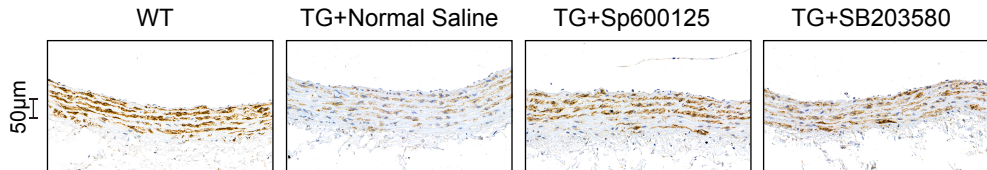

Supplement: Supplementary Materials — Supplementary material 1: sequence of plasmid pcDNA3.1(+)_myc-His A- L3MBTL4. Supplementary material 2: designed gene-specific primers. Figure S1: pathological changes of the myocardium and kidney in L3MBTL4 TGs were improved when inhibiting p38 and JNK. Figure S2: p38/JNK was essential for elevated proliferative activity and depressed apoptotic status of smooth muscle cells in aortic tissue. Figure S3: L3MBTL4 and its downstream p38 and JNK were partly mediated by TIMP-1/ MMP-9 and TIMP-2/MMP-2. Figure S4: p38/JNK inhibition lead to improved expression of VSMCs differentiation markers in L3MBTL4 TGs. [file 3123968.f1.zip › 3123968.f1/R1-FigureS4.pdf]
